# Navigating Digital Security and Usability Challenges for Older Adults With Cognitive Concerns

**DOI:** 10.1093/geroni/igaf122.580

**Published:** 2025-12-31

**Authors:** Debaleena Chattopadhyay, Tasneem Mubashshira

**Affiliations:** University of Illinois Chicago, Chicago, Illinois, United States; University of Illinois Chicago, Chicago, Illinois, United States

## Abstract

Emerging technologies—including smartphone apps, smart home devices, digital health services, and wearables—offer opportunities to support older adults, particularly those at risk for Alzheimer’s Disease and Alzheimer’s Disease Related Dementias (AD/ADRD), in maintaining health and independence. As digital tools become integral to instrumental activities of daily living (I/ADLs)—such as medication management, transportation, and telehealth—effective use becomes increasingly critical. However, cognitive changes can limit the breadth of technology use even after initial adoption. In 2024, we conducted ten mobile technology learning workshops with community-dwelling older adults in greater Chicago (median age = 74). Sixty-five percent of participants reported subjective cognitive decline (SCD), assessed using the Cognitive Function Instrument. SCD is increasingly recognized as a potential early marker of non-normative cognitive decline and ADRD progression. A reflexive thematic analysis of workshop recordings revealed a persistent tension between security and usability. Participants expressed heightened anxiety about making mistakes or compromising personal information, leading to technology avoidance—even when they were interested. At the same time, they struggled with managing authentication credentials and resisted software updates, fearing disruptions to familiarity and ease of use. Current security measures—such as frequent password changes, multi-factor authentication, and mandatory updates—exacerbate these barriers, further limiting digital engagement. To facilitate technology use among older adults at risk for AD/ADRD, technology design must balance security with cognitive accessibility. Addressing these challenges is critical for fostering digital inclusion and supporting the functional independence of older adults with emerging cognitive concerns.

